# 1,2-Dihydro-9*H*-carbazole-4(3*H*)-thione

**DOI:** 10.1107/S1600536811006623

**Published:** 2011-03-02

**Authors:** W. Adam Phelan, Maria Ngu-Schwemlein, Frank R. Fronczek, Mark L. McLaughlin, Steven F. Watkins

**Affiliations:** a Department of Chemistry, Louisiana State University, Baton Rouge, LA 70803, USA

## Abstract

The crystal structure of the title compound, C_12_H_11_NS, features parallel chains of alternating N—H⋯S hydrogen-bonded mirror-image conformers along [10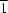]. The mol­ecular conformation is that of an envelope, with all of the framework atoms except one close to a mean plane (rms deviation 0.054 Å); one C atom of the cyclo­hexene­thione ring forms the envelope flap, which makes a dihedral angle of 48.6 (1)° with the rest of the mol­ecule. There is a π–π* inter­action between pairs of enanti­omers in adjacent chains; the distance between parallel planes is 3.466 (1) Å.

## Related literature

For related structures, see: Hökelek *et al.* (1998 ▶); Ianelli *et al.* (1994 ▶); Çaylak *et al.* (2007 ▶); Rodriguez *et al.* (1989 ▶). Hückel calculations were performed using *Chem3DPro* (Cambridgesoft, 2009 ▶).
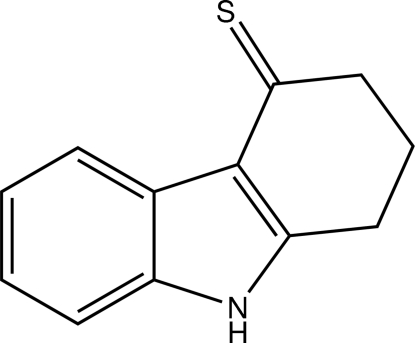


## Experimental

### 

#### Crystal data

C_12_H_11_NS
*M* *_r_* = 201.28
Monoclinic, 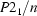

*a* = 8.6353 (14) Å
*b* = 12.1395 (15) Å
*c* = 9.5808 (14) Å
β = 104.599 (10)°
*V* = 971.9 (2) Å^3^
*Z* = 4
Mo *K*α radiation
μ = 0.29 mm^−1^
*T* = 90 K
0.38 × 0.33 × 0.15 mm

#### Data collection

Nonius KappaCCD diffractometer
Absorption correction: multi-scan (*SCALEPACK*; Otwinowski & Minor, 1997 ▶) *T*_min_ = 0.900, *T*_max_ = 0.958
6145 measured reflections
3305 independent reflections
2915 reflections with *I* > 2σ(*I*)
*R*_int_ = 0.018

#### Refinement

*R*[*F* ^2^ > 2σ(*F* ^2^)] = 0.033
*wR*(*F* ^2^) = 0.087
*S* = 1.04
3305 reflections
128 parameters
H-atom parameters constrained
Δρ_max_ = 0.42 e Å^−3^
Δρ_min_ = −0.30 e Å^−3^

### 

Data collection: *COLLECT* (Nonius, 2000 ▶); cell refinement: *SCALEPACK* (Otwinowski & Minor, 1997 ▶); data reduction: *DENZO* (Otwinowski & Minor, 1997 ▶) and *SCALEPACK*; program(s) used to solve structure: *SHELXS97* (Sheldrick, 2008 ▶); program(s) used to refine structure: *SHELXL97* (Sheldrick, 2008 ▶); molecular graphics: *ORTEP-3 for Windows* (Farrugia, 1997 ▶); software used to prepare material for publication: *WinGX* (Farrugia, 1999 ▶).

## Supplementary Material

Crystal structure: contains datablocks global, I. DOI: 10.1107/S1600536811006623/fl2336sup1.cif

Structure factors: contains datablocks I. DOI: 10.1107/S1600536811006623/fl2336Isup2.hkl

Additional supplementary materials:  crystallographic information; 3D view; checkCIF report

## Figures and Tables

**Table 1:** Hydrogen-bond geometry (Å, °)

*D*—H⋯*A*	*D*—H	H⋯*A*	*D*⋯*A*	*D*—H⋯*A*
N9—H9⋯S1^i^	0.88	2.45	3.3187 (9)	172

Symmetry code: (i) 
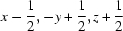
.

**Fig. 1.**
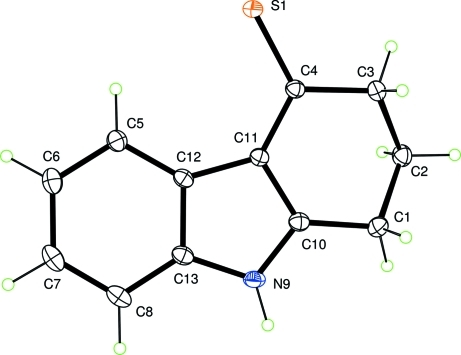
View of 1 (50% probability displacement ellipsoids).

**Fig. 2.**
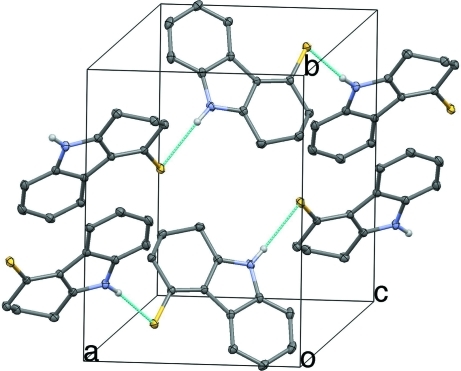
The unit cell, illustrating hydrogen bonds.

## supplementary crystallographic information

### Comment

The title compound (1, Fig.1) is the sulfur analog of substituted carbazole 1,2,3-trihydrocarbazol-4(9*H*)-one (2) (Rodriguez *et al.*, 1989). Both 1 and 2 show the same molecular conformation (envelope, with flap angles 48.6 (1)° for 1 and 48.2 (1)° for 2) and similar H-bonded chains (Table 1) of alternating enantiomers (N···S = 3.319 (1) Å, N—H···S = 172.0 (1)°, and N···S═C = 98.0 (1)° for 1, N···O = 2.829 (1) Å, N—H···O = 162.3 (1)° and H···O═C = 117.5 (1)° for 2).

In 1, all H-bonded chains are parallel, extending along the [101] crystallographic direction, and adjacent chains 5.583 (1) Å apart are arranged in corrugated sheets parallel to the (010) crystallographic plane (Fig.  22)). The mean planes of adjacent sheets are 5.099 (1) Å apart, but enantiomers in adjacent sheets have parallel π-nodal planes and are only 3.466 (1) Å apart, indicative of a π-π* interaction. Extended Hückel calculations (*Chem3DPro*, Cambridgesoft, 2009) suggest that the π-HOMO and π*-LUMO orbitals in 1 are larger and closer in energy than those in 2. This may explain why molecules of 2 show no π-type interaction and are thus packed in a different pattern: H-bonded chains 5.359 (1) Å apart extend along the [011] and [011] directions in alternating sheets, so adjacent sheets are rotated by 76.5 (1)°. The distance between adjacent sheets is 4.979 (1) Å and the only interactions between them are C—H···C van der Waals and C—H···O contacts.

### Experimental

A solution of 1,2-dihydrocarbazol-4(3*H*)-one (5.4 mmol) in anhydrous 1,2-dimethoxyethane (30 ml) was stirred at room temperature for 15 min. Upon dissolution, the solution was chilled in an ice-water bath. Lawesson reagent, 2,4-bis(4-methoxyphenyl)-1,3,2,4-dithiadiphosphetane 2,4-disulfide, (2.9 mmol) was added to the vigorously stirred cold solution. The resulting mixture was stirred for 5 min and then allowed to warm to room temperature. After stirring for an additional 10 min, the white suspension dissolved, and the reaction mixture turned deep orange. The reaction mixture was poured into 150 ml of chilled water and the orange suspension was extracted with CHCl_3_ (3 *x* 80 ml). Evaporation under reduced pressure left a deep orange residue, which was purified on a silica column (100 g). The orange band was eluted with ethyl acetate. Evaporation of the solvent *in vacuo* gave the title compound as a yellow powder (92%). Recrystallization from dichloromethane yielded yellow needles, m.p. 173–175°C.

### Refinement

All H atoms were placed in calculated positions, guided by difference maps, with C—H bond distances 0.95–0.99 Å, N—H 0.88 Å, *U*_iso_=1.2*U*_eq_, and thereafter refined as riding.

### Crystal data

**Table:**

C_12_H_11_NS	*F*(000) = 424
*M**_r_* = 201.28	*D*_x_ = 1.376 Mg m^−^^3^
Monoclinic, *P*2_1_/*n*	Melting point: 447(1) K
Hall symbol: -P 2yn	Mo *K*α radiation, λ = 0.71073 Å
*a* = 8.6353 (14) Å	Cell parameters from 3145 reflections
*b* = 12.1395 (15) Å	θ = 2.8–31.8°
*c* = 9.5808 (14) Å	µ = 0.29 mm^−^^1^
β = 104.599 (10)°	*T* = 90 K
*V* = 971.9 (2) Å^3^	Prism, yellow
*Z* = 4	0.38 × 0.33 × 0.15 mm

### Data collection

**Table:**

Nonius KappaCCD diffractometer	2915 reflections with *I* > 2σ(*I*)
ω and φ scans	*R*_int_ = 0.018
Absorption correction: multi-scan (*SCALEPACK*; Otwinowski & Minor, 1997)	θ_max_ = 31.8°, θ_min_ = 2.8°
*T*_min_ = 0.900, *T*_max_ = 0.958	*h* = −12→12
6145 measured reflections	*k* = −17→15
3305 independent reflections	*l* = −14→14

### Refinement

**Table:**

Refinement on *F*^2^	H-atom parameters constrained
Least-squares matrix: full	*w* = 1/[σ^2^(*F*_o_^2^) + (0.0381*P*)^2^ + 0.4607*P*] where *P* = (*F*_o_^2^ + 2*F*_c_^2^)/3
*R*[*F*^2^ > 2σ(*F*^2^)] = 0.033	(Δ/σ)_max_ = 0.001
*wR*(*F*^2^) = 0.087	Δρ_max_ = 0.42 e Å^−^^3^
*S* = 1.03	Δρ_min_ = −0.30 e Å^−^^3^
3305 reflections	Extinction correction: *SHELXL97* (Sheldrick, 2008), Fc^*^=kFc[1+0.001xFc^2^λ^3^/sin(2θ)]^-1/4^
128 parameters	Extinction coefficient: 0.007 (2)
0 restraints	

### Special details

**Table:**

Geometry. All e.s.d.'s (except the e.s.d. in the dihedral angle between two l.s. planes) are estimated using the full covariance matrix. The cell e.s.d.'s are taken into account individually in the estimation of e.s.d.'s in distances, angles and torsion angles; correlations between e.s.d.'s in cell parameters are only used when they are defined by crystal symmetry. An approximate (isotropic) treatment of cell e.s.d.'s is used for estimating e.s.d.'s involving l.s. planes.

### Fractional atomic coordinates and isotropic or equivalent isotropic displacement parameters (Å^2^)

**Table:**

	*x*	*y*	*z*	*U*_iso_*/*U*_eq_	
S1	0.73572 (3)	0.09251 (2)	0.17793 (3)	0.01632 (8)	
N9	0.40190 (10)	0.23264 (7)	0.49231 (9)	0.01459 (16)	
H9	0.3596	0.2739	0.5486	0.018*	
C1	0.64126 (13)	0.35282 (8)	0.50486 (11)	0.01640 (19)	
H1A	0.6442	0.3693	0.6067	0.02*	
H1B	0.5975	0.4178	0.4456	0.02*	
C2	0.81014 (12)	0.32729 (9)	0.49014 (11)	0.01613 (19)	
H2A	0.8612	0.2724	0.5639	0.019*	
H2B	0.8756	0.3953	0.5072	0.019*	
C3	0.80512 (12)	0.28219 (8)	0.33970 (11)	0.01504 (18)	
H3A	0.766	0.3409	0.2677	0.018*	
H3B	0.9153	0.2631	0.3355	0.018*	
C4	0.69952 (12)	0.18152 (8)	0.29869 (10)	0.01239 (17)	
C5	0.40380 (12)	−0.00895 (8)	0.27655 (10)	0.01451 (18)	
H5	0.4724	−0.0398	0.2234	0.017*	
C6	0.26491 (13)	−0.06343 (9)	0.28577 (11)	0.0179 (2)	
H6	0.2394	−0.1325	0.2389	0.021*	
C7	0.16174 (13)	−0.01841 (9)	0.36297 (12)	0.0196 (2)	
H7	0.0665	−0.0567	0.3655	0.024*	
C8	0.19647 (13)	0.08102 (9)	0.43570 (11)	0.0181 (2)	
H8	0.1273	0.1116	0.4885	0.022*	
C10	0.53890 (12)	0.25494 (8)	0.45510 (10)	0.01313 (17)	
C11	0.56930 (11)	0.17216 (8)	0.36283 (10)	0.01191 (17)	
C12	0.44039 (11)	0.09230 (8)	0.34726 (10)	0.01234 (17)	
C13	0.33703 (12)	0.13394 (8)	0.42784 (10)	0.01382 (18)	

### Atomic displacement parameters (Å^2^)

**Table:**

	*U*^11^	*U*^22^	*U*^33^	*U*^12^	*U*^13^	*U*^23^
S1	0.01819 (13)	0.01449 (12)	0.01981 (13)	−0.00184 (8)	0.01132 (9)	−0.00327 (8)
N9	0.0156 (4)	0.0158 (4)	0.0144 (4)	0.0018 (3)	0.0077 (3)	−0.0002 (3)
C1	0.0194 (5)	0.0149 (4)	0.0159 (4)	−0.0014 (4)	0.0063 (4)	−0.0029 (3)
C2	0.0162 (4)	0.0168 (4)	0.0150 (4)	−0.0032 (4)	0.0031 (3)	−0.0016 (3)
C3	0.0149 (4)	0.0145 (4)	0.0167 (4)	−0.0031 (3)	0.0059 (3)	−0.0012 (3)
C4	0.0128 (4)	0.0125 (4)	0.0123 (4)	0.0008 (3)	0.0041 (3)	0.0011 (3)
C5	0.0149 (4)	0.0145 (4)	0.0141 (4)	−0.0005 (3)	0.0035 (3)	0.0017 (3)
C6	0.0182 (5)	0.0169 (5)	0.0175 (4)	−0.0039 (4)	0.0027 (4)	0.0026 (4)
C7	0.0156 (5)	0.0230 (5)	0.0204 (5)	−0.0045 (4)	0.0049 (4)	0.0056 (4)
C8	0.0140 (4)	0.0235 (5)	0.0182 (4)	0.0001 (4)	0.0068 (4)	0.0046 (4)
C10	0.0140 (4)	0.0142 (4)	0.0115 (4)	0.0012 (3)	0.0039 (3)	0.0012 (3)
C11	0.0120 (4)	0.0126 (4)	0.0115 (4)	0.0001 (3)	0.0037 (3)	0.0005 (3)
C12	0.0117 (4)	0.0142 (4)	0.0115 (4)	0.0006 (3)	0.0035 (3)	0.0026 (3)
C13	0.0136 (4)	0.0160 (4)	0.0126 (4)	0.0011 (3)	0.0046 (3)	0.0026 (3)

### Geometric parameters (Å, °)

**Table:**

C1—C10	1.4860 (14)	C5—H5	0.95
C1—C2	1.5318 (14)	C6—C7	1.4042 (16)
C1—H1A	0.99	C6—H6	0.95
C1—H1B	0.99	C7—C8	1.3882 (16)
C2—C3	1.5322 (14)	C7—H7	0.95
C2—H2A	0.99	C8—C13	1.3920 (14)
C2—H2B	0.99	C8—H8	0.95
C3—C4	1.5162 (14)	C10—N9	1.3463 (12)
C3—H3A	0.99	C10—C11	1.4061 (13)
C3—H3B	0.99	C11—C12	1.4553 (13)
C4—C11	1.4154 (13)	C12—C13	1.4127 (13)
C4—S1	1.6692 (10)	C13—N9	1.3988 (13)
C5—C6	1.3917 (14)	N9—H9	0.88
C5—C12	1.4006 (13)		
			
C10—C1—C2	108.17 (8)	C5—C6—C7	121.32 (10)
C10—C1—H1A	110.1	C5—C6—H6	119.3
C2—C1—H1A	110.1	C7—C6—H6	119.3
C10—C1—H1B	110.1	C8—C7—C6	121.15 (10)
C2—C1—H1B	110.1	C8—C7—H7	119.4
H1A—C1—H1B	108.4	C6—C7—H7	119.4
C1—C2—C3	110.92 (8)	C7—C8—C13	117.08 (10)
C1—C2—H2A	109.5	C7—C8—H8	121.5
C3—C2—H2A	109.5	C13—C8—H8	121.5
C1—C2—H2B	109.5	N9—C10—C11	109.79 (9)
C3—C2—H2B	109.5	N9—C10—C1	124.55 (9)
H2A—C2—H2B	108	C11—C10—C1	125.66 (9)
C4—C3—C2	113.85 (8)	C10—C11—C4	120.68 (9)
C4—C3—H3A	108.8	C10—C11—C12	106.34 (8)
C2—C3—H3A	108.8	C4—C11—C12	132.83 (9)
C4—C3—H3B	108.8	C5—C12—C13	118.80 (9)
C2—C3—H3B	108.8	C5—C12—C11	135.05 (9)
H3A—C3—H3B	107.7	C13—C12—C11	106.14 (8)
C11—C4—C3	116.35 (8)	C8—C13—N9	128.98 (9)
C11—C4—S1	123.85 (7)	C8—C13—C12	122.96 (10)
C3—C4—S1	119.73 (7)	N9—C13—C12	108.06 (8)
C6—C5—C12	118.64 (9)	C10—N9—C13	109.64 (8)
C6—C5—H5	120.7	C10—N9—H9	125.2
C12—C5—H5	120.7	C13—N9—H9	125.2
			
C10—C1—C2—C3	49.84 (11)	C6—C5—C12—C13	1.45 (14)
C1—C2—C3—C4	−55.67 (11)	C6—C5—C12—C11	−179.79 (10)
C2—C3—C4—C11	28.23 (12)	C10—C11—C12—C5	−177.03 (10)
C2—C3—C4—S1	−154.52 (8)	C4—C11—C12—C5	7.46 (19)
C12—C5—C6—C7	0.52 (15)	C10—C11—C12—C13	1.84 (10)
C5—C6—C7—C8	−1.47 (16)	C4—C11—C12—C13	−173.67 (10)
C6—C7—C8—C13	0.35 (15)	C7—C8—C13—N9	−178.60 (10)
C2—C1—C10—N9	158.35 (9)	C7—C8—C13—C12	1.70 (15)
C2—C1—C10—C11	−21.72 (13)	C5—C12—C13—C8	−2.64 (14)
N9—C10—C11—C4	174.61 (9)	C11—C12—C13—C8	178.27 (9)
C1—C10—C11—C4	−5.33 (15)	C5—C12—C13—N9	177.61 (8)
N9—C10—C11—C12	−1.56 (11)	C11—C12—C13—N9	−1.47 (10)
C1—C10—C11—C12	178.50 (9)	C11—C10—N9—C13	0.66 (11)
C3—C4—C11—C10	2.22 (13)	C1—C10—N9—C13	−179.40 (9)
S1—C4—C11—C10	−174.91 (7)	C8—C13—N9—C10	−179.18 (10)
C3—C4—C11—C12	177.20 (10)	C12—C13—N9—C10	0.55 (11)
S1—C4—C11—C12	0.08 (16)		

### Hydrogen-bond geometry (Å, °)

**Table:**

*D*—H···*A*	*D*—H	H···*A*	*D*···*A*	*D*—H···*A*
N9—H9···S1^i^	0.88	2.45	3.3187 (9)	172

Symmetry codes: (i) *x*−1/2, −*y*+1/2, *z*+1/2.
